# Editorial: Research on value development in the school context: new directions and approaches from an international perspective

**DOI:** 10.1007/s10212-025-01003-y

**Published:** 2025-10-28

**Authors:** Elena Makarova, Anna K. Döring, Ella Daniel, Maya Benish-Weisman

**Affiliations:** 1 Institute for Educational Sciences, University of Basel, Hofackerstrasse 30, 4132 Muttenz, Switzerland; 2 Psychology, School of Social Sciences, University of Westminster, 115 New Cavendish Street, London, W1W 6UW UK; 3 Department of School Counseling and Special Education, Tel Aviv University, Ramat Aviv, 6997801 Tel Aviv, Israel; 4 Paul Baerwald School of Social Work and Social Welfare, The Hebrew University of Jerusalem, Jerusalem, Israel

**Keywords:** Value development, Values education, Value formation, Human values, School

## Abstract

This special issue advances the study of value formation in educational settings by introducing and applying the *Value Transmission in the School Context* model, which has been developed on the basis of a conceptual synthesis of Schwartz’s Theory of Basic Human Values, Bronfenbrenner’s Ecological Systems Theory, and Fend’s New Theory of School. While human values are central to global educational agendas, empirical research on how values develop in school contexts remains limited. The ten original studies in this issue draw on diverse methodologies and data from six countries to examine value development in children and adolescents across micro-, meso-, and macro-levels of schooling. Together, the contributions highlight the dynamic interplay between vertical value transmission (from educational systems to classroom practices and vice versa) and horizontal transmission (between teachers and students, as well as among peers). The findings emphasize that schools are not only institutional settings for value education but also social spaces where values are continuously shaped through interaction and negotiation. This editorial situates the studies within the proposed framework offering a cohesive perspective on the multilevel processes that shape value development in schools. It concludes by identifying key directions for future research that account for systemic structures, developmental dynamics, and contextual factors.

## Introduction

Human values have long been central to education worldwide, shaping the agendas of supranational organizations such as the Organisation for Economic Co-operation and Development (OECD) and the Council of Europe (CE). As defined by the Council of Europe, “values are general beliefs that individuals hold about the desirable goals that should be striven for in life. They motivate action and they also serve as guiding principles for deciding how to act” (Council of Europe, 2016, p. 36). Values are also integral to the OECD’s *Learning Compass 2030* (2019), outlining the humanistic values students need to navigate in order to contribute to society responsibly. Similarly, the Council of Europe’s *Reference Framework of Competences for a Democratic Culture* (RFCDC) (2016) defines key values necessary for active participation in democratic societies. Those include universalism values such as human dignity, human rights, cultural diversity, democracy, justice, fairness, equality, as well as self-direction values that emphasize self-awareness and critical thinking. Consequently, in the European educational landscape, fostering human values is seen as essential to strengthening democratic culture and guiding individual and collective action. However, despite their inclusion in educational curricula, research on children’s value formation within the school context remains limited.

At the same time, advancements in social and developmental psychology have provided new insights into the nature and development of values during childhood and adolescence (Daniel et al., 2020; Döring et al., 2016, 2017). Schwartz’s *Theory of Basic Human Values* (Schwartz, 1992) has played a key role in shaping research on value priorities across cultures and age groups (Daniel & Benish-Weisman, 2019; Döring et al., 2010, 2016) and in examining the influence of values on attitudes and behaviors among children and adolescents (Abramson et al., 2018; Benish-Weisman, 2019; McDonald et al., 2015). This theory offers a structured framework for understanding value conflicts and cultural variations. Yet, despite these theoretical advancements, their application to the school context remains underexplored.

Preliminary research suggests that Schwartz’s *Theory of Basic Human Values* has significant potential for educational psychology (Berson & Oreg, 2016; Daniel et al., 2013; Hadar & Benish-Weisman, 2019). Recent studies (Oeschger et al., 2022; Scholz-Kuhn et al., 2023) demonstrate the growing availability of high-quality data that could support further investigation. Following the momentum of a special section in *Social Development* edited by Döring et al. (2016), this special issue aims to advance the study of value development in the school context.

The present special issue brings together, for the first time, ten original studies that investigate the role of schools in shaping value development across different educational contexts. The contributions employ diverse methodologies, including cross-sectional and longitudinal studies, social network analyses, mixed-methods approaches, and qualitative research, offering a comprehensive examination of value formation in childhood and adolescence. This methodological variety enhances our understanding of how values relate to social behavior, peer interactions, academic choices, and well-being in educational settings.

The studies span multiple cultural and national contexts, including Switzerland, Poland, Italy, Israel, and the UK, demonstrating the broad relevance of values education across different education systems. Some contributions focus on early childhood, examining how values relate to prosocial and antisocial behaviors, while others investigate value development in adolescence, exploring its links to peer socialization, academic pathways, and digital interventions. Additionally, several studies highlight the role of teachers, the school climate, and institutional frameworks in the process of students’ value formation. All studies are conceptually situated within *Schwartz's* *Theory of Basic Human* *Values*. The use of a single theory across the different studies, despite their variability in methodology, cultural context, level of analysis, and age group, is a unique strength of this special issue. Previous studies often relied on conceptualizations of values unique to this study, or they each applied different values or different definitions, and thus, it was more difficult to form a complete picture.

Overall, this editorial aims to situate these contributions conceptually by developing a comprehensive theoretical model to research value formation within the school context and engaging with the broader academic discourse on value transmission in education. By synthesizing key findings, it provides a cohesive perspective on the mechanisms, contexts, and implications of value formation in schools, guiding future research and educational practice in this critical area.

## Theoretical foundations of value development

### Schwartz’s theory of basic Human values as a developmental framework

**Fig. 1:**
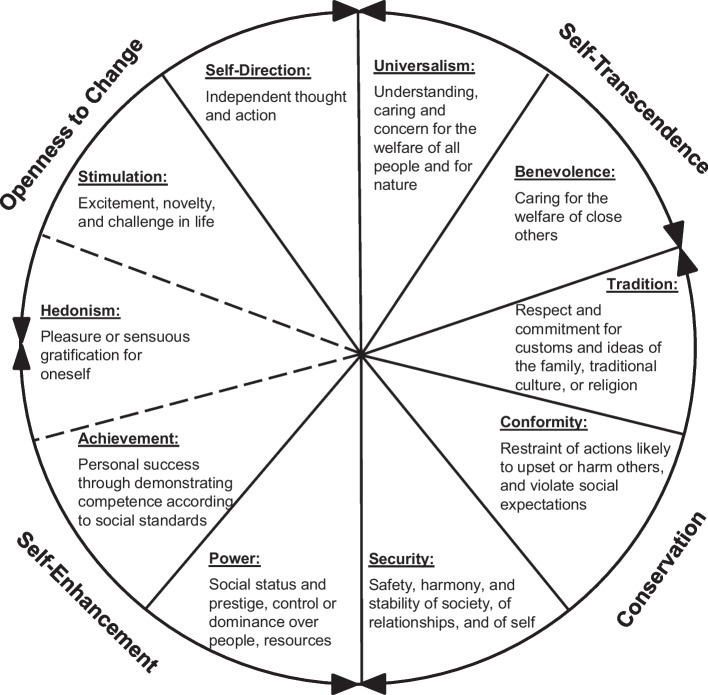
Schwartz’ (1992) model of human values

This special issue is rooted in the most researched theory of human values, as developed by Schwartz (1992, 1994), which has been confirmed in hundreds of studies around the globe (Schwartz, 2012). Schwartz defined values as broad life goals that serve as guiding principles in a person’s life. As such, values are relatively stable across situations and across time. Schwartz and collaborators (reviewed in Sagiv et al., 2017) found that personal values can be organized in a circular structure (Fig. 1). In this structure, single values are subsumed under the heading of either of ten basic values: *universalism*, *benevolence*, *tradition*, *conformity*, *security*, *power*, *achievement*, *hedonism*, *stimulation*, and *self-direction*. These basic values are arranged alongside a circular continuum, wherein neighboring values have a similar motivational goal, whereas opposing values have conflicting motivational goals. For example, values of universalism and benevolence share a motivation to support and help others and are compatible in this respect. But the pursuit of these *self-transcendence values* potentially conflicts with the pursuit of the opposed *self-enhancement values* (power and achievement), as these are targeted at obtaining prestige, control, and success for oneself. In addition to these two opposing poles of self-transcendence versus self-enhancement, the model presents a second dimension, which is composed of the poles *openness to change* (self-direction, stimulation, and sometimes hedonism values, therefore the dotted line in Fig. 1) versus *conservation* (tradition, conformity, and security values). Because of this structure, values that relate positively to one value tend to also relate positively to neighboring values, and to relate negatively to conflicting values. Hence, this structure illustrates why, for example, it may be challenging to encourage children to excel in class (self-enhancement) while also promoting kindness among them (self-transcendence), as these values can lead to conflict. However, it is easier to encourage children to be kind to one another while also mastering the challenges of learning and understanding (openness to change).

Applying the framework of Schwartz’s Theory of Basic Human Values as a developmental framework, Döring and colleagues (2010) developed and validated the Picture-Based Value Survey for Children (PBVS-C) for assessing values in children (Döring et al., 2010). In addition, Collins and colleagues (2017) validated the Animated Values Instrument (AVI), a multi-sensory instrument designed to assess young children’s values using a best–worst scaling approach (Lee et al., 2019). These developments to assess values directly from children enabled revealing that children as young as five years have a clear and differentiated understanding of human values (Collins et al., 2017; Döring et al., 2016; Elizarov et al., 2023), and that their value priorities are relatively stable over time (Cieciuch et al., 2016; Daniel et al., 2024a) and predict children’s behavior longitudinally (Benish-Weisman, 2015; Berson & Oreg, 2016; Daniel et al., 2024a; Vecchione et al., 2016).

### A contextual approach to value formation

The formation of children’s values takes place in a variety of social contexts. One influential theoretical framework for understanding this process is Bronfenbrenner’s *Ecological Systems Theory* (Bronfenbrenner, 2005; Bronfenbrenner & Morris, 2006). This theory conceptualizes individual development within interrelated social environments and is operationalized through the PPCT model: Person, Process, Context, and Time. The *person a*spect describes the characteristics of the developing child (e.g. age, sex, ethnicity); *Proximal processes*, capture reciprocal interactions (e.g., interactions with peers, teachers); the *context aspect* includes four nested and interrelated systems: (1) the micro-system in which the child is an active member (e.g., home, school, peers); (2) the mesosystem at the intersection of two or more micro-systems; (3) the exosystems, which are proximal contexts, in which the child is not an active member (e.g., parents’ workplace); and (4) the macro-system (e.g., culture, society) are more distal contexts, that encompass the mesosystems and exosystems. Finally, *time* (the chronosystem) captures how the timing and patterning of events—both in the individual’s life course and in historical time—influence development.

Among these social environments, both *family* (Döring et al., 2017; Makarova et al., 2018) and *school* play central roles in value formation. While the family is considered a primary agent of socialization, *schools act as secondary socialization instances* (Tillmann, 2020) with substantial societal relevance in the transmission of values (Matthes, 2004). Unlike the informal character of family interactions, schools provide a *formalized, institutionalized,* and *legally sanctioned framework* for children’s value development (Standop, 2013), facilitating the connection between individuals, groups, and society (Zajda, 2014).

#### School as a societal institution of value formation

According to the *New Theory of School* proposed by Fend (2008a) schools fulfill four key societal functions such as *enculturation* (i.e., schools transmit cultural knowledge, values, and norms, fostering identity formation and social cohesion), *qualification* (i.e., schools equip individuals with the necessary skills and competencies for participation in the labor market and economic system), *allocation* (i.e., schools serve as mechanisms for social stratification by distributing individuals into different career paths and social positions based on their academic performance and acquired certificates), and *integration* (i.e., schools contribute to social integration by fostering a sense of belonging, societal participation, and shared democratic values, mitigating social inequalities). In the context of value formation among children and youth, enculturation fosters the internalization of fundamental values such as moral responsibility, while integration promotes social cohesion by reinforcing societally shared values and norms. Finally, value transmission also plays a prominent role within the functions of qualification and allocation, as it involves values which are relevant to academic achievement and further career paths.

In addition, Fend (2008b) conceptually describes how cultural content is transmitted through the education system, suggesting a multi-stage transformation process across different system levels. At the *macro-level*, policy decisions shape curricula, timetables, and educational pathways. The *meso-level* involves translating institutional requirements into local school environments through value transmission. At the *micro-level*, teachers reinterpret, adapt, and implement this content in the classroom according to its specific requirements. It suggests that schools facilitate the internalization of culturally shared values, social norms and rules, often embedded in the curriculum as value-related teaching objectives (Halstead, 1996; Lovat, 2011; Oeschger et al., 2022; Powney et al., 1995), playing a crucial role in integrating young people into society by conveying prosocial values essential for responsible citizenship (Berson & Oreg, 2016; Scholz-Kuhn et al., 2021).

#### Value formation in classrooms

Within classrooms, value transmission is embedded in complex social dynamics. At the system level, classroom interaction begins with reciprocal perception and double contingency, where each person’s actions are influenced by and in turn influence the other’s action, which forms the basis for the construction of teaching and learning as a social system. Since teaching situations arise from communication that cannot be unilaterally controlled, symmetry—mutual engagement and communicative reciprocity—becomes central (Herzog, 2002, p. 454). In this context, both teachers and peers function as socializing agents, conveying values through continuous interactions. The teacher plays a key role in modeling and mediating values, drawing on both their personal value system and their socialization values—the values they aim to transmit to students (Auer et al., 2023; Benish-Weisman et al., 2013; Knafo & Galansky, 2008). These systems may overlap but also diverge, particularly in culturally diverse classrooms where differences may arise between the values of teachers, schools, and students’ home environments (Knafo, 2003; Tam & Lee, 2010; Whitbeck & Gecas, 1988).

At the same time, *peers* contribute significantly to value development within this system of classroom interaction. Especially in adolescence, when distancing from parental norms becomes developmentally relevant, peer groups gain increasing importance in shaping values and behaviors (Benish-Weisman et al., 2022; Branje, 2018; Kindschi et al., 2019). Through joint activities, negotiations, and mutual recognition, peers engage in value-laden exchanges that challenge and refine individual perspectives (Benish-Weisman, 2024). Already in primary school, as children develop greater perspective-taking abilities, peer influence becomes more pronounced (Knafo-Noam et al., 2024), contributing to a dynamic and interactive process of value formation within the classroom microculture (Benish-Weisman et al., 2022).

At the *didactic level*—the complexity of the social situation is functionally reduced, and asymmetry becomes more prominent. Here, roles are institutionally framed: teachers are responsible for instruction, while students engage in learning. Actions at this level can be categorized as individual (e.g., the teacher’s instructional strategies, students’ learning activities) or social (e.g., maintaining order, group tasks), engaging all classroom participants (Herzog, 2002, p. 454). At this level, teachers deliberately communicate and convey their values through pedagogical choices and practices. This includes their classroom management strategies (Barni et al., 2018; Cadima et al., 2015), their intentional value-oriented educational and socialization goals (Auer et al., 2023; Tamm et al., 2020), and their broader selection of educational aims (Leenders et al., 2008). Teachers may also serve as role models at the system level, influencing students’ value development through processes such as modeling and identification, as proposed by Bardi and Goodwin (2011).

#### A holistic model of value transmission within the school context

**Fig. 2:**
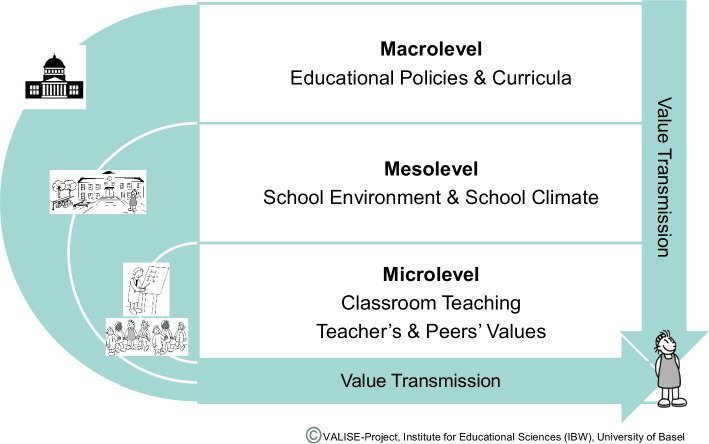
Value transmission in the school context: levels and dimensions

Bringing together the theoretical and empirical evidence on value formation within the school context, we introduce a novel systematic conceptualization of *Value Transmission within the School Context*. This theoretical model portrays the formation of children’s values along two dimensions: (a) a *vertical dimension* which subsumes all mechanisms of value transmission from the macro-system (educational system) through the mesosystem (the school environment) to the micro-system (processes within the classroom) including feedback loops that influence value transmission in the opposite direction—from micro- to meso- and macro-levels; and (b) a *horizontal dimension* which encompasses processes of value transmission within the classroom (micro-system) between teachers and students or among peers (Fig. 2).

At the center of the model is *the individual child with their values*. Recent studies across cultures have shown that already at the beginning of elementary school, children’s value structures reflect the motivational compatibilities and conflicts in Schwartz’s model and are almost as differentiated as value structures in adolescence and adulthood (Döring et al., 2015, 2016). Throughout the elementary school years, the individual child’s value structure tends to show an increasingly fine-grained differentiation between values, which coincides with cognitive maturation and the child’s understanding of values as abstract concepts (Daniel et al., 2023; Knafo-Noam et al., 2024; Misgav & Daniel, 2022; Shachnai & Daniel, 2020). In the transition from childhood to adolescence, and in the educational systems from elementary to secondary school, longitudinal studies have depicted the emergence of incoherence within value structures (Shachnai & Daniel, 2020; Daniel et al, 2023). Incoherence in value structures in turn was found to predict the strength of value change in the following years (Daniel et al., 2024a). Value change across the school years follows systematic patterns of development. For example, Cieciuch et al. (2016) studied value priorities of Polish primary school children who were between seven and ten years old at the first point of measurement over two years and found moderate stability with self-transcendence values being most and self-enhancement values being least important, but change towards the adolescent years with values of openness to change becoming more and values of conservation becoming less important. Similar patterns of change were identified in an Israeli sample (Daniel et al., 2024a). Recent research has identified a range of key factors that drive this change in an interaction of characteristics of the individual (e.g., genes, Twito & Knafo-Noam, 2020), specific life experiences (e.g., living in a war zone; Bardi & Goodwin, 2011; Daniel et al., 2013), and environments the child grows up in (e.g., a religious family, Döring et al., 2016). Importantly, the individual child’s values systematically predict the child’s actions (Abramson et al., 2018; Benish-Weisman, 2015, 2019), whereby values of self-transcendence relate to prosocial and compassionate behavior while values of self-enhancement relate to competitive and sometimes also aggressive behavior. Similarly, values of openness to change relate to academic performance and scientific reasoning skills (Elizarov et al., 2025) and self-directed behavior whereas values of conservation relate to disciplined behavior (Berson & Oreg, 2016).

This dynamic and systematic change of children’s values and their behavior has been portrayed in the micro-system classroom in schools in Switzerland (Kindschi et al., 2019; Scholz-Kuhn et al., 2023), Poland (Kindschi et al., 2019), Germany (Döring & Hillbrink, 2015), Italy (Barni et al., 2018; Vecchione et al., 2016), and Israel (Benish-Weisman et al., 2015; Elizarov et al., 2024a). Recognizing that a single action can be motivated by a range of different values as well as by demands of the situation and context, research of values-behavior relationships has focused on ‘value-expressive behaviors’—specific actions that tend to be substantially motivated by specific values (Bardi & Schwartz, 2003; Berson & Oreg, 2016). Examples of value-expressive behavior in the classroom are helping a classmate (self-transcendence), working hard to get the best grades in class (self-enhancement), abiding by school rules (conservation), or exploring unconventional ideas (openness to change) (Vecchione et al., 2016). In this vein, Scholz-Kuhn and colleagues (2023) showed systematic relationships between children’s values and their value-expressive behavior as rated by their classroom teacher, with supportive behavior (e.g., “is sensitive to other children’s needs”) going along with self-transcendence values, achievement-oriented behavior (e.g., “is very competitive in class”) going along with self-enhancement values, disciplined behavior (e.g., “obeys the rules in class”) going along with conservation values and learning-oriented behavior (e.g., “asks many good questions in class”) going along with openness to change values. Studies based on self-reported behavior (Vecchione et al., 2016) as well as behavior rated by classmates (Benish-Weisman, 2015; Daniel et al., 2020), or teachers (Elizarov et al., 2024a) have confirmed these patterns longitudinally, where values predicted future behavior and (to a smaller extent) behavior predicted future values (Vecchione et al., 2016).

Within the micro-system classroom, the classroom teacher as well as peers (the child’s classmates) are key actors. Teachers bring to the classroom their own personal values as well as their socialization values (i.e., the values they would like their pupils to endorse), both of which relate to teachers’ classroom management style (authoritarian, authoritative, permissive, Barni et al., 2018). Peers contribute significantly to pupils’ values—an effect that has been studied primarily in adolescence. As Benish-Weisman (2024) phrased it: “Tell me who your friends are, and I will tell you who you are.” In a large longitudinal dataset including data from 15,008 children from grade three to nine, Benish-Weisman and colleagues (2022) found that the values of peers in the classroom correlated positively with the strengthening of children’s corresponding values and can have an indirect effect on children’s behavior. Kindschi and colleagues (2019) studied the effect of peers in network analyses in 73 classrooms in Switzerland and Poland and found evidence of external validation through social comparison, where adolescents shifted the emphasis they placed on values toward the average expression of their friends. When studying stability and change of values in the classroom, the vast majority of studies to date have observed processes over time. However, Döring and Hillbrink (2015) have demonstrated that a classroom-based intervention can initiate value change: German adolescents in the experimental group watched excerpts from the movie ‘Into the wild’, after which value changed in the direction of those displayed by the movie’s protagonist with a significant increase in the importance assigned to universalism and a significant decrease in the importance assigned to conformity as compared to the control group.

At the meso-level, we observe the micro-systems—the classrooms and their key actors—interact, where research highlights the impact of the school environment and school climate. Caprara and colleagues (2014) developed and implemented an intervention to promote prosocial behavior and values in Italian middle schools, with the values-based curriculum implemented across the school. Following the intervention, they found an increase in helping behavior and a decrease in physical and verbal aggression in the intervention group as compared to the control group, as well as higher grades at the end of middle school.

School values are an important aspect of the school climate (Daniel et al., 2013; Hofman-Towfigh, 2007). In view of Schwartz’s (1992) model of human values, research has identified school climate scales that map onto the four higher-order values, with climate of support mapping onto self-transcendence, climate of performance mapping onto self-enhancement, climate of stability mapping onto conservation, and climate of innovation mapping onto openness to change values (Berson & Oreg, 2016). A key actor at the school level is the head-teacher, who significantly shapes the school climate and, in turn, affects pupils’ values and behavior (Berson & Oreg, 2016).

At the macro-level, values are embedded in educational policies and curricula. For example, Oeschger and colleagues (2022) conducted a structuring content analysis of the primary school curriculum, which applies in all 21 German-speaking cantons in Switzerland and found a strong focus on values of self-direction, stimulation (e.g., curiosity), and universalism. Importantly, this curriculum was developed pre-pandemic, and when key values from the curriculum were presented to Swiss primary school teachers during the pandemic, they placed more importance on values of conformity and security than the analysis of the curriculum suggested. This shows the importance of considering the chronosystem, the situation of school values within the historical context. In the macro-system, studies also show differences between children’s and adolescents’ values in different schools (Auer et al., 2023; Knafo et al., 2008), between values that prevail at home and in the child’s school (Knafo, 2003), and between teachers’ values in different countries (Oeschger et al., 2024).

## Empirical contributions of the special issue: dynamic interplay of actors and pathways of value transmission in the school context

The studies of the present special issue are situated within the theoretical model of *Value Transmission in the School Context*. They provide a multifaceted exploration of value development by analyzing effects and dynamics of value transmission in the school context, based on data from elementary and secondary schools in Australia, Switzerland, Israel, Italy, Poland, and the UK.

### Value transmission at the micro-, meso-, and macro-level of the school context

At the *micro-level*, studies elaborate on children’s values within the context of the education system. First, the studies elucidate the importance of values in promoting social behaviors, and well-being. Elizarov et al. (2024b) show that self-transcendence values in kindergarten positively predict prosocial and negatively predict antisocial behaviors, providing insights into value-behavior relations at a very early age. This study further sheds light on the socio-cognitive factors underlying these relations, as the value behavior relations are mediated by social information processing patterns. Scholz-Kuhn et al. (2025) portray developmental trajectories of children’s personal values and their behavior in the classroom at the start of primary school, showing a substantial increase in the importance children assign to self-transcendence and a decrease in the importance children assign to self-enhancement values. Highlighting the importance of researching variation of values within and between classrooms and introducing the classroom as a key level of analysis, they demonstrate bidirectional longitudinal relationships between behaviors and values in early elementary school. Collins et al.’s (2024) study researches elementary school children’s healthy versus unhealthy values that promote or hamper (respectively) subjective well-being. They also link growth values to well-being and anxiety values to lower social support, particularly for girls. Finally, the studies look for the root of values in relationships within the school context, including relationships with peers and teachers. Focusing on peer relationships, Cieciuch et al. (2024) employed a longitudinal social network approach in secondary school classrooms and provided evidence for peer selection and socialization effects, showing that adolescents develop values through friendship and trust networks. While Cieciuch et al. researched how value change occurs naturally over time, Russo et al. (2023) designed a web-based intervention aimed to initiate value change and foster prosocial values in secondary school students through priming, consistency maintenance, and direct persuasion. In a mixed-methods quasi-experimental study conducted during the online teaching stage of the COVID-19 pandemic, they found a significant increase in the importance of conservation, but not self-transcendence values, in the intervention but not the control group, where students in the experimental group highlighted the importance of prosocial action in light of the isolation and safeguarding brought by the pandemic.

At the *meso-level*, studies underscore the interaction of teachers’ goals, school structures, school ethos, and climate in shaping students’ values. Segal et al. (2025) explore how secondary school students’ personal values as well as their value-based academic goals relate to their academic track choices, showing that openness to change is linked to selecting exact sciences, while self-transcendence values are more common among psychology students. Döring et al. (2024) gave voice to educators through interviewing elementary school teachers and uncovered all of Schwartz’s values in the interview data. They further revealed how teachers aim to shape students’ values through modeling, explicit guidance, and through providing opportunities for behavior. They showcase how value transmission occurs in taught lessons as well as in the wider school environment and how each school’s culture and ethos play vital roles. Oeschger et al. (2024) highlight the reciprocal longitudinal relationship between elementary school teachers’ value-related educational goals (i.e., the values they would like to see in their students) and the climate they perceived in their school. Being historically situated during the COVID-19 pandemic, they found that a school climate of innovation positively predicted the teachers’ goals of openness to change at the onset of the pandemic, whereas the opposite prediction (from teachers’ goals to school climate) occurred at later time points. Similarly, a school climate of stability predicted teachers’ conservation goals. In addition, a study by Vaknin & Schachter (2024) in Israeli religious high schools show that students from less-observant Masorti families perceive school climate less positively and identify less with school values than their peers from Orthodox families, with identity-related climate factors mediating this effect and highlighting the importance of pedagogical support for identity development in fostering adolescents’ value formation.

At the *macro-level*, studies examine how educational structures and institutional narratives relate to value formation. In this special issue, Daniel et al. (2024b) analyze school vision statements as the aspired future states of the school and show how institutional priorities reflect broader societal values, depicting both shared value hierarchies as well as variation between different school types, public-religious and public schools.

### The dynamic interplay of value transmission processes between the micro-, meso-, and macro-level

In addition to portraying value transmission processes at the micro-, meso, or macro-level, the evidence in this special issue also shows interactions between the three levels and vertical value transmission:

Establishing interactions of processes within and between classrooms, Scholz Kuhn et al. (2025) demonstrated that, at the beginning of primary school, children’s value priorities (how important they find each value) and the way children act on their values (their value-expressive behavior) differed substantially within as well as between the 96 classrooms investigated. While children’s value priorities changed dynamically over the first two years of their school journey, the trajectory of behavior that their classroom teacher observed remained relatively flat, with no significant change at the classroom level. In the same vein, Cieciuch et al. (2024) portrayed networks of friendship, advice, and trust longitudinally in classrooms, starting at the onset of secondary school. While the specific structure of the network differed between the 34 classrooms investigated, the study found support for both selection as well as socialization effects across classrooms, meaning that children chose to become friends with, sought advice from, and trusted classmates whose values were similar to their own (selection), and children’s values became more similar to their classmates’ values as well (socialization). While Scholz-Kuhn et al. (2025) and Cieciuch et al. (2024) explored value formation as it occurred, Russo et al. (2023) demonstrated that an intervention that was implemented at school and classroom level could change individual adolescents’ values. Investigating the reverse direction, Segal et al. (2025) showed that adolescents’ values were related to which of the academic tracks provided by their school they chose. Finally, Vaknin & Schachter (2024) broaden the scope beyond the school as they examine identity-related school climate and demonstrate that value incongruence between the home and the school is related to students’ identification with school values.

Establishing interactions between the classroom and school level, Oeschger et al.’s (2024) longitudinal study revealed processes in two directions, where (1) the school climate predicted primary school teachers’ value-related educational goals over time (top down) and (2) primary school teachers’ educational values predicted the school climate (bottom up). Importantly, this study as well as Russo et al.’s (2023) study suggest that the chronosystem (i.e., the historical context) affects processes at micro-, meso-, and macro-level, as the COVID-19 pandemic led to an increase in the importance of conservation and a decrease in the importance of openness to change values at all levels and possibly stronger top-down processes at work at the beginning of the pandemic, with bottom-up processes becoming more influential again as security measures that had been implemented during the pandemic being lifted. Finally, the qualitative interview study by Döring et al. (2024) shows that opportunities for value formation can occur in the classroom as well as the wider school environment and are shaped by educational goals of the classroom teacher, the headteacher, as well as guidelines from the national curriculum.

*Overall*, these findings emphasize that schools serve as key agents of value education and socialization, highlighting that children’s value formation is shaped by multiple interrelated processes of vertical and horizontal value transmission, as proposed in the *Value Transmission in the School Context* model. Moreover, the evidence presented in this special issue goes beyond understanding value transmission within isolated micro-, meso-, or macro-systems. Instead, the studies illustrate the dynamic interplay of processes both within and across these systems in diverse and nuanced ways.

## Future directions for research on values in the school context

The contributions in this special issue provide empirical evidence for the multilevel processes that shape children’s value development in schools. Guided by the *Value Transmission in the School Context* model, future research should continue to investigate mechanisms of value formation at the micro-, meso-, and macro-levels, with particular attention to their interactions over time and across cultural contexts.

*At the micro-level*, further research is needed to understand how children and adolescents internalize values through their everyday interactions with teachers and peers. Longitudinal studies could investigate how value development unfolds during key educational transitions and how individual differences interact with classroom experiences. In particular, more work is needed to understand how students navigate competing value messages and develop strategies of selective internalization or resistance. Moreover, classroom-based studies should examine how pedagogical practices as well as peer relationships and dynamics contribute to the reinforcement or transformation of personal values over time.

*At the meso-level*, future studies could apply mixed-method approaches and explore how the broader school environment shapes value development through school climate, organizational culture, and structural arrangements. This includes examining how institutional factors—such as school leadership, pedagogical models, or teacher collaboration—facilitate or hinder coherent value socialization in schools. In addition, there is a need to understand how schools can create shared value orientations and how a possible value-related misalignment is negotiated between schools and families.

*At the macro-level*, studies could further investigate how educational systems, national curricula, and broader societal discourses inform the values promoted in schools. This includes exploring how shifts in education policy, global crises, or sociopolitical debates shape the priorities embedded in institutional narratives such as school mission statements. Comparative and cross-cultural research designs can provide insight into how structural conditions and cultural contexts influence the alignment—or dissonance—between policy-level and value agendas of schools and educators. Such work is essential for understanding how schools function as both agents and arenas of societal value transmission.

As a whole, these future directions point to the importance of integrated, multilevel approaches to value development in schools. Advancing this field requires sustained attention to the dynamic interplay between individual agency, institutional culture, and systemic structures.

## Conclusions

In conclusion, the *Value Transmission in the School Context* model offers a comprehensive framework for understanding how values are shaped and transmitted within educational settings. By integrating both vertical and horizontal dimensions, this model captures the interplay between systemic influences and interpersonal interactions, highlighting the crucial role of schools, teachers, and peers in the development of children’s values. This conceptualization not only deepens our theoretical understanding of value formation but also provides a foundation for future research and educational practices aimed at fostering children’s value formation.

## Data Availability

Not applicable.
